# Green extraction of lutein from marigold flower petals, process optimization and its potential to improve the oxidative stability of sunflower oil

**DOI:** 10.1016/j.ultsonch.2022.105994

**Published:** 2022-04-01

**Authors:** Shaziya Manzoor, Rubiya Rashid, Bibhu Prasad Panda, Vasudha Sharma, Mohd Azhar

**Affiliations:** a Department of Food Science and Technology, University of Kashmir, Srinagar, J&K, India; b Jamia Hamdard, New Delhi, India

**Keywords:** *Tagetes erecta*, Antioxidant, Optimization, Chromatography, Saponification

## Abstract

Marigold flower petals are considered the richest source of lutein which possesses immense applications in the food and health sector. The study was undertaken to improve the stability of sunflower oil by enriching it with lutein extracted from marigold flower petals using safe and green technology. The extraction of lutein was optimized using Box-Behnken design by ultrasound-assisted extraction (UAE) employing sunflower oil as a solvent. The impact of three independent variables i.e., ultrasonic intensity, solid to solvent ratio, and extraction time were evaluated on the amount of lutein extracted and its antioxidant activity. Highest amount of lutein (21.23 mg/g) was extracted by employing ultrasonic intensity of 70 W/m2, extraction time of 12.5 min, and solid to solvent ratio of 15.75%. FT-IR spectra of lutein extracted by ultrasound and conventional extraction show similar peaks depicting that ultrasound does not have any impact on the functionality of lutein. Sunflower oil incorporated with lutein at 1000 PPM and the synthetic antioxidant (TBHQ) showed good oxidative stability than oil with 500 PPM lutein and no lutein during accelerated storage for a month. The oxidative stability was shown by different oil samples in the following order: TBHQ = 1000PPM lutein˃500PPM lutein ˃control oil. It was concluded that the ultrasound technique extracts lutein efficiently from marigold flowers and this lutein was effective in improving the oxidative stability of sunflower oil under accelerated storage conditions.

## 1 Introduction

Marigold (*Tagetes spp*.), is a prominent decorative plant that is spread all over the world with a diverse range of species. The two most important species cultivated in India include *Tagetes erecta* and *Tagetes patula* with three general varieties of red, yellow, and orange coloured flowers [1]. Marigold flower petals are found to be the richest source of polyphenols [2] and carotenoids out of which lutein esters comprise 70–79% of the total carotenoids [3], [4]. The increased attention paid by researchers towards this ornamental plant is because of its immense medicinal potential owing to its bioactive constituents, particularly in minimizing the chances of macular degeneration [5], [6]. Lutein has also been well documented for its antioxidant and anti-inflammatory potential, neuroprotective effects, and prevention against cardiovascular disease. Owing to its numerous benefits lutein finds widespread usage in food and pharmaceutical industries where it is marketed in the form of capsules, nutraceuticals [7], and food colourant [8], etc.

Lutein has been extracted from marigold petals using both conventional(simple maceration, soxhlet extraction) and novel methods (supercritical fluid extraction enzyme-assisted extraction, microwave-assisted extraction [9], [10]. However conventional methods are equipped with enormous disadvantages like longer extraction times, high quantity of solvents used and heat degradation of carotenoids[11]. To overcome this challenge, several steps have been taken by researchers to ensure greener, sustainable and viable techniques for lutein extraction. Extraction using ultrasound is one such novel technology, offering several advantages compared to other methods such as higher extraction yield, decreased time for extraction, less consumption of energy[12], [13].

Extracting carotenoid compounds by the use of edible oils fulfils all the approaches to be considered as a green process [14]. The advantage of using oil as an extracting solvent is that carotenoids possess higher solubility in edible oils due to their lipophilic nature and they also prevent the degradation of carotenoids by acting as a barrier to oxygen[15]. Furthermore, no requirement of carotenoid and oil separation is there, as the carotenoid enriched oil finds application as a carotenoid source in aquaculture related feeds [16], as a natural colourant in fish sausage[17] and bakery [18], etc. UAE combined with edible oil is reported to enhance the process efficiency in every possible way making the extraction process fully environment friendly. Various studies have been conducted so far for the carotenoid extraction using UAE with edible oil as an extraction solvent like extraction of carotenoid lycopene from tomato waste using sunflower oil [19], pomegranate carotenoids using soyabean oil and sunflower oil[20], orange peel carotenoids using olive oil [21], etc. Extraction of lutein using edible oils is not much reported. To name a few, studies of [22] Gao et al., (2009) and (2010)[23] where they utilized edible oil as a cosolvent for lutein ester extraction from Chinese marigold using supercritical fluid extraction.

In this study we aim to optimize the process of extraction of lutein in sunflower oil from marigold flower petals by using ultrasonication. The potential of lutein to stabilize the sunflower oil was also analysed and compared with synthetic antioxidant (TBHQ) during the accelerated storage conditions. This study finds its applications in the stabilization of various oils and the formation of healthy foods using lutein enriched oils.

## 2 Experimental

### 2.1 Materials and methods

Pusa Naringi Gainda (PNG) (*Tagetes erecta*) variety of Indian marigold was procured from Indian Agricultural Research Institute (IARI) Pusa, New Delhi, India. The moisture content of fresh marigold flower petals was reduced from 75% to 4.9% using a lyophilizer (Buchi lyovapor L-200, Switzerland). The dried material was then grinded in a laboratory grinder and the fraction was passed through a 60-mesh sieve to obtain a reduced particle size. Collected powder was then stored in vacuum-sealed opaque bags at 4 °C until use. To prevent light degradation of the carotenoids, this experiment was conducted in a dark space. Sunflower oil was chosen for lutein extraction because of its ability to extract the higher content of carotenoids as compared to other vegetable oils[20]. Sunflower oil was purchased from a local departmental store. Lutein standard and all other analytical grade chemicals were procured from Sigma Aldrich (New Delhi). All the experiments were performed in accordance with relevant guidelines and regulations.

### 2.2 Ultrasound -assisted extraction using sunflower oil

**Instrumentation and method.** UAE was conducted by using a probe sonicator (Q125-QSONICA, USA) with Ti-Al-V sonoprobe (13 mm), 125 W of nominal output power and frequency of 20 kHz. Direct immersion of probe into the sample solution facilitates achievement of higher power in a shorter duration. The ultrasonication was carried out in pulsed mode to avoid excessive heating. Vibrations at the probe were controlled by the digital amplitude control of the processor, which allows the probe to get fixed at any level ranging from 20 to 100% of nominal power.

Powdered PNG petals were mixed with 150 mL edible oil. During this whole process, the sample was kept in temperature regulated water bath. The extracts obtained were then centrifuged at 1500 g for 10 min to remove coarse impurities before lutein analysis.

**Experimental design**. Box–Behnken design (BBD) was implemented to check the influence of various independent factors like ultrasonic intensity (50–90 W/m^2^), extraction time (5–20 min), and solid to solvent ratio (1.5–30% w/v) on the amount of lutein extracted from marigold petals and its antioxidant activity; and optimize the process parameters for lutein extraction. The BBD was preferred due to fewer runs required by it as compared to central composite design where three or four variables are intended for study. The independent variables chosen were ultrasonic intensity (A), extraction time (B), solid to solvent ratio (C). Three varying independent parameters encrypted to three levels (−1, 0, and 1) **(**supplementary Table) and 17 experimental runs (Table 1) measured in a randomized order were conducted to maximize the response value. Regression analysis was then performed and a polynomial equation (2nd order) was obtained which explains the correlation among the measured responses and the independent variables.

$$Y=\beta_{0}+\sum_{i=1}^{k}\beta_{i}X_{i}+\sum_{i=1}^{k}\beta_{\mathrm{ii}}Xi^{2}+\sum_{i=j}^{k}\beta_{\mathrm{ij}}X_{i}X_{j}\;\;\;\;\;\;\;\;\;\;\;\;\;\;\;\;\;\;\;\;\;\;\;\;\;\;\;\;\;\;\;\;\;\;\;\;\;Eq\;1$$

**Table 1:** Box-Behnken Design matrix and experimentally obtained results of investigated responses.

	**Independent variables**			**Responses**	
**Run**	**A:** **ultrasonic power(watt)**	**B:** **Time (Minutes)**	**C:** **Solid/solvent (%)**	**Amount of lutein(mg/g)**	**Antioxidant** **activity (%)**
1	70	12.5	15.75	21.23	91.05
2	70	12.5	15.75	21.05	90.62
3	50	20	15.75	10.63	74.95
4	70	20	1.5	14.76	84.39
5	70	20	30	16.01	86.71
6	70	5	1.5	10.04	78.66
7	70	5	30	11.81	80.93
8	70	12.5	15.75	21.03	92.87
9	90	5	15.75	9.54	76.34
10	90	12.5	1.5	11.15	77.45
11	70	12.5	15.75	21.06	90.05
12	50	12.5	30	8.91	70.54
13	50	5	15.75	7.14	66.17
14	70	12.5	15.75	20.02	90.32
15	50	12.5	1.5	8.85	67.85
16	90	12.5	30	13.95	79.65
17 Predicted value Experimental value	90 70.00 70.00	20 12.50 12.50	15.75 15.75 15.75	10.99 20.87 21.23 ± 0.89^a^	73.94 90.98 90.32 ± 1.68^b^

Where Y represents the measured responses (Lutein content and antioxidant activity), Xi and Xj represent the independent variables (i ≠ j, i and j range from 1 to k), and k represents the number of independent parameters (k = 3). β0, βi, βii and βij refer to regression coefficients for intercept, linear, quadratic and interaction terms, respectively.

### 2.3 Conventional solvent extraction (CSE)

Lutein was extracted by the method followed by (Sachindra & Mahendrekar.,2005)[24] with slight modifications. In brief extraction was carried out by blending 1 g of powdered PNG petals with a mixture of 200 mL acetone and petroleum ether (50% v/v) for 24 h in a shaking water bath at 40 °C. 0.1% NaCl solution was used to wash the mixture to separate phases and remove traces of petroleum ether. The mixture was then centrifuged (4000 g, 25 °C) for 15 min. The supernatant obtained was freeze-dried in a lyophilizer. Dried samples were kept at −20 °C prior to analysis. This whole procedure was performed under dark conditions to prevent light degradation and isomerization of lutein.

### 2.4 Total flavonoids (TFs)

TFs of UAE extract and CSE extract of PNG petals were analysed following the aluminium chloride colorimetric method (Chang et al., 2002). In short, 1 mL of extract was taken in a flask and mixed with 4 mL of water. After 5 min, 0.3 mL of 5% sodium nitrate and 0.3 mL of 10% Aluminium chloride was added. After incubation of the above mixture at room temperature for 10 min,1 mL of NaOH was added (1 M) and volume was makeup to 10 mL by adding water. The absorbance of the mixture against the blank was taken at 510 nm. TFs were determined from the 5-point calibration curve of quercetin (y = 0.0035x + 0.0813) with r^2^ = 0.9744, where y represents sample absorbance and × indicate the sample concentration, from 10 to 70 μg/mL. The total flavonoids were calculated in terms of quercetin equivalent (mg QE/g) per g of dry weight.

### 2.5 De-esterification of lutein

Free lutein was obtained by converting the lutein fatty acid esters by the process of saponification following the method of Boonnoun et al., (2012)[25]. Briefly,1g of obtained oleoresin was added to the mixture of 0.6 g of KOH and 10 mL ethanol and was kept in a shaking water bath at 250 rpm and 50 °C for 5hrs. Ethanol (50 mL), 5% Na_2_SO_4_ (100 mL), and diethyl ether (80 mL) were then added to the mixture in a separating funnel. The upper phase containing free lutein stock solution was collected; while a lower transparent phase was disposed of. Free lutein obtained was then dried in a freeze drier and stored at −20 °C for further analysis.

### 2.6 Quantification and identification of lutein using high performance thin layer chromatography (HPTLC)

**Fig. 1:**
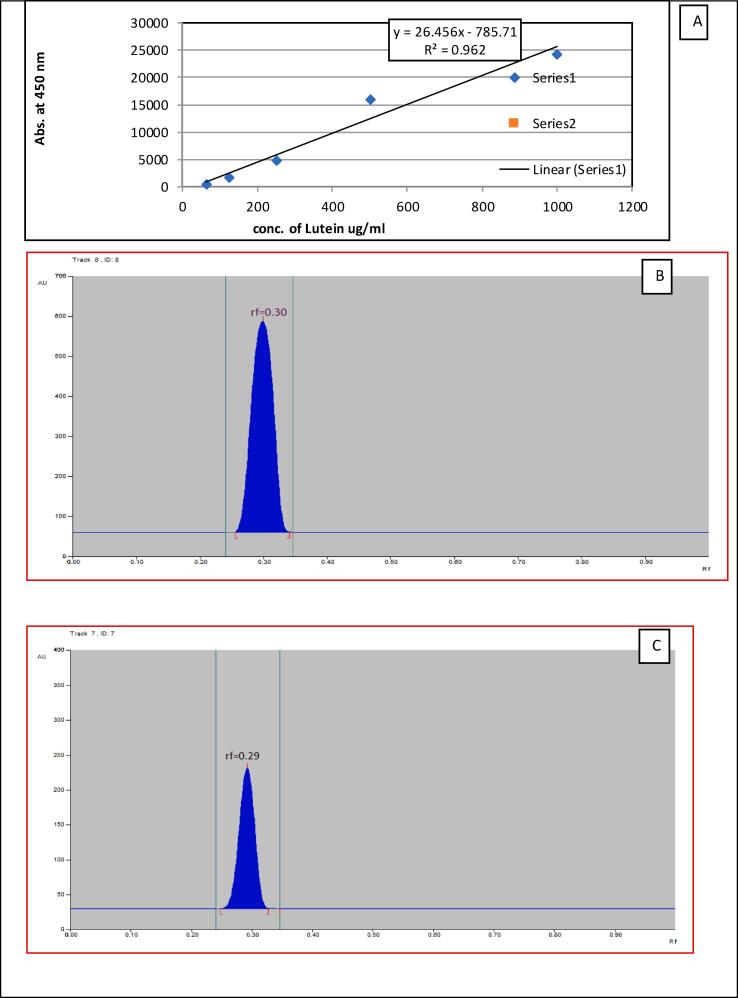
**HPTLC of lutein extracted from PNG** (A) 5-point standard calibration curve and (B) HPTLC graph of lutein standard and (C)PNG lutein developed by hexane: ethyl acetate (70:30v/v) detected at a wavelength of 450 nm (Retention factor = 0.30).

HPTLC analysis was conducted by CAMAG HPTLC system (CAMAG, Switzerland) integrated with automatic sample applicator, TLC scanner with CATS software. The sample was applied on the silica gel pre-coated plate having a uniform thickness of 0.2 mm. Prior to HPTLC analysis, the TLC plate was activated by washing it with methanol and then subsequently dried at 110 °C for 10 min. After plate activation, both sample and standard spots were applied on it (maintaining a gap of 10 mm between 2 bands) using a 20 μL syringe with an automatic sample applicator under the stream of N_2_ gas. For the development of chromatogram, these plates were then dipped in a twin trough chamber filled with Hexane: ethyl acetate (70:30 v/v) mobile phase. Plate was removed from the chamber once the mobile phase reaches up to the distance of 9 cm from the base. After drying it for 15 min, it was scanned at 450 nm by TLC scanner-3. Lutein was analyzed in samples by comparing it with the retention time of standard and quantified by a five-point calibration curve of different concentrations [26] (Fig. 1).

### 2.7 FTIR-spectra of lutein

The nature of Lutein extracted by UAE and CSE was analyzed with the help of FTIR (Cary 630 FTIR, Agilent Technologies, USA) in the wavelength range of 400 to 4000 cm^−1^. FTIR spectrum was obtained at the resolution of 4 cm through pro-software version 2.2.5 (Agilent Technologies, USA).

### 2.8 Antioxidant activity

#### 2.8.1 DPPH (2,2′-diphenyl-1-picrylhydrazyl radical) assay

The antioxidant activity of the extracts was determined by DPPH assay following the method of Brand-Williams et al., (1995)[27]. In brief, 1 mL sample solution was blended with 1 mL of 0.2 mili-Molar methanolic solution of DPPH. The mixture obtained was shaken vigorously and then kept for 30 min at room temperature for incubation. Absorbance was taken at 517 nm using a UV–visible spectrophotometer (Shimadzu UV-1700, Tokyo, Japan). DPPH solution without the addition of oil sample was taken as control. The percentage of free radicals scavenged by DPPH radical was calculated using the below equation.

(2) $$\%Inhibition=\frac{A_{C0-A_{\mathrm{Sa}}}}{A_{\mathrm{Co}}}\times 100$$

As_a_ and Ac_o_ represent the absorbance of the sample and control respectively.

#### 2.8.2 Ferric reducing antioxidant power (FRAP):

The antioxidant activity of lutein obtained under the optimum conditions was further investigated by FRAP and ABTS assay. FRAP assay was carried out according to the methods detailed by Benzie & Strain (1996). The FRAP working reagent consisted of 300 mM acetate buffer (pH 3.6), 20 mM FeCl3 solution and 10 mM TPTZ solution in 40 mM HCl in the ratio of 10:1:1 (v/v), respectively. 10 μL sample extract was mixed with 1.8 mL of FRAP stock solution and the mixture was incubated at room temperature in darkness for 10 min. After incubation, absorbance of the mixture was taken at 593 nm. The activities were represented in terms of mmol of trolox/g dry marigold petal.

#### 2.8.3 2,2-azinobis- (3 ethylbenzothiazoline-6-sulfonate (ABTS) assay

The ABTS assay was done following the method of Sharma et al. (2008), with slight modifications. An ABTS•+ stock solution was prepared by diluting the ABTS•+ stock solution with ethanol to get an absorbance of 0.70 ± 0.02 at 734 nm. Breifly,10 μL aliquots of the sample extracts were mixed with 200 μL ABTS•+ working solution and the mixture was allowed to react at 25 °C in darkness for 10 min. The absorbance of the mixture was then recorded and the results were expressed as mmol of trolox/g dry marigold petal.

### 2.9 Oxidative stability analysis

The impact of lutein on oxidative stability of sunflower oil under accelerated storage conditions (50 °C for 4 weeks) was evaluated. Two varying concentrations of lutein:500 ppm and 1000 ppm/litre were added into the sunflower oil and was compared against the oxidative stability of synthetic antioxidant, TBHQ (200 ppm). Sunflower oil without any antioxidant was taken as control. After sample preparation, 30 mL of each oil sample was stored in amber-colored bottles and was kept in the oven at 50 °C for 4 weeks. Oxidative stability of stored oils was checked every 7 days.

#### 2.9.1 Acid value

The acid value was evaluated following the official method AOCS (Cd 3d-63). Briefly 2.5 mL of oil samples was mixed with 50 mL of ethanol and 1 mL phenolphthalein indicator in a conical flask. The mixture was heated at 80 °C for about 12 min in a water bath. This was followed by titration of the solution against the 0.1 N NaOH. The acid value was expressed in terms of mg KOH/g of test portion and calculated by using the below mentioned equation:

Acid value = (56.1*V*N)/W (3).

where, V represents volume of NaOH used, N represents normality of NaOH and W represents weight of test sample.

#### 2.9.2 Peroxide value (PV)

The peroxide value was carried out by following the official method of AOCS Cd 8–53. Briefly 5 g of oil samples was dissolved in the mixture of chloroform and glacial acetic acid (2:3) and 0.5 mL of saturated potassium iodide solution was added to the mixture. The mixture was left to react properly by keeping it in dark for 30 min. The free iodine was titrated against 0.1 N of sodium thiosulphate solution using starch solution (1 mL of 1%) as an indicator. The PV value (mEq of oxygen/Kg of oil) was determined by using the following equation:

Peroxide value=(S-B) *W*N (4).

where B = volume of sodium thiosulphate used for blank, S = volume of sodium thiosulphate consumed by test oil sample, W = weight of sample and N = normality of standard sodium thiosulphate.

#### 2.9.3 p-anisidine value

p-Anisidine value of oil samples was done according to official method AOCS Cd 18–90. Firstly,1g of test oil sample was taken and diluted with 25 mL of isooctane. The absorbance of the mixture was recorded at 350 nm using a spectrophotometer (Eppendorf Biospectrometer). Then 5 mL oil sample was mixed with 1 mL of p-anisidine reagent (0.25 g p-anisidine in 100 mL of glacial acetic acid) and absorbance was recorded at 350 nm. Mixture of 5 mL isooctane and 1 mL of p-anisidine reagent was taken as blank. The p-anisidine value was determined by using following the equation.

p-anisidine = [25*(1.2As-Ab)]/W (5).

where, As indicates Absorbance of oil samples with p-anisidine reagent, Ab indicates Absorbance of oil and W represents weight of oil sample.

#### 2.9.4 Conjugated dienes and trienes

Conjugated dienes and trienes were evaluated following the method of Li, et al (2021)[28]. Oil samples (50 µL) was dissolved in isooctane and measured at 232 for dienes and 270 nm for trienes using UV spectrophotometer (Eppendorf Biospectrometer). Conjugated dienes and trienes were determined using the below equation.

(6) $$E_{1cm\lambda }^{1\%}=\frac{A\lambda }{\omega }$$

Where A(λ) represents the absorption of sample at 232 nm and 270 nm; and ω reflects concentration of test sample (g/100 mL).

The effect of ultrasound on oxidative stability of sunflower oil was evaluated by differentiating the oxidative stability parameters between untreated control oil and ultrasound treated oil at optimum conditions.

**Statistical analysis*.*** Statistical BBD applied to our study was generated and evaluated using Design Expert 12 software (Stat-Ease, Inc., USA). Statistical analysis of the design and the determination of interaction between the variables and their effect on responses were investigated with the help of ANOVA The adequacy of the applied model and statistical significance of its parameters was assessed by regression coefficient (R^2^), F test, and p values. Response surface plots were assessed for the respective interaction between 2 variables and their impact on measured response keeping the third variable constant.

## 3 Results and discussions

### 3.1 Box-Behnken design of experiments

#### 3.1.1 Model adequacy

The regression coefficients determined by ANOVA of the fitted model for the interaction variables (ultrasonic intensity, extraction time, and solid/solvent ratio) and the various statistical parameters like F-values, coefficient of determination (R^2^), lack of fit (p-value) are some of the important parameters which define the adequacy of the model (Table 2, Table 3). The influence of variable parameters on measured responses in the fitted model is only significant if it is having a *p*-value of<0.05, which in this case comes out to be < 0.0001, making this model a robust one. The coefficient of determination (R^2^) for both the responses was greater than 0.98 indicating a higher accuracy of the fitted model. A higher F value (Table 2) further proves the significance of model [29].

**Table 2:** Analysis of variance (ANOVA) of the fitted models for total lutein content and antioxidant activity.

**Source**	**Sum of squares**	**D.F**	**Mean square**	**F-value**	**P-value**	**R^2^**	**Remarks**
**Lutein content (mg/g)**							
Model	404.95	9	44.99	56.74	<0.0001	0.98	significant
residual	5.55	1	0.7930				
Lack of fit	4.61	7	1.54	6.49	0.0512		non-significant
Pure error	0.9459	3	0.2365				
Corrected total	410.50	4					
**Antioxidant activity (%)**							
Model	1156.73	9	128.53	45.65	<0.0001	0.98	significant
residual	19.71	7	2.82				
Lack of fit	14.70	3	4.90	3.91	0.1102		non-significant
Pure error	5.01	4	1.25				
Corrected total	1176.44	16

**Table 3:** Estimated regression coefficients for total lutein content and antioxidant activity.

**Factor**	**Lutein content(mg/g)**				**Antioxidant activity (%)**			
	Coefficient estimate	Standard error	F-value	P-value	Coefficient estimate	Standard error	F-value	P-value
Intercept	20.88	0.3983			90.98	0.7504		
A	1.26	0.3148	16.08	0.0051	3.48	0.5932	34.49	0.0006
B	1.73	0.3148	30.28	0.0009	2.24	0.5932	14.21	0.0070
C	0.7350	0.3148	5.45	0.0523	1.19	0.5932	3.99	0.0859
AB	−0.5100	0.4453	1.31	0.2897	−2.80	0.8390	11.10	0.0126
AC	0.6850	0.4453	2.37	0.1678	−0.1225	0.8390	0.0213	0.8880
BC	−0.1300	0.4453	0.0852	0.7788	0.0125	0.8390	0.0002	0.9885
A^2^	−6.87	0.4340	250.70	< 0.0001	−13.47	0.8177	271.18	< 0.0001
B^2^	−4.43	0.4340	104.27	< 0.0001	−4.67	0.8177	32.56	0.0007
C^2^	−3.29	0.4340	57.52	0.0001	−3.64	0.8177	19.85	0.0030

All the three independent variables that are ultrasonic intensity (A), time (B), and the ratio of solid to solvent (C) imposed a dominant influence on lutein extraction and its antioxidant activity. However, ultrasonic intensity and extraction time were considered as highly influencing parameters on both the responses as shown by their p-values (0.0006 and 0.0070) respectively, whereas solid/solvent ratio seemed less significant.

### 3.2 Optimization using UAE with sunflower oil

**Fig. 2:**
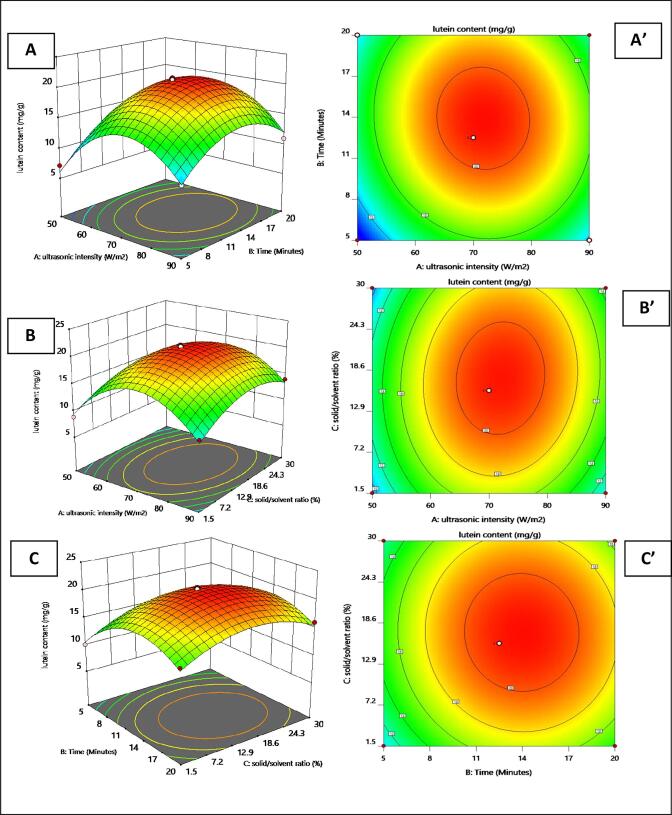
**3D Response plots and 2D contour plots of lutein content** Footnote: 3D response surface plots (A, B, C) and 2D contour plots (A’, B’, C’) of the lutein content(mg/g) as function of ultrasonic intensity/m^2^, extraction time(minutes) and solid/solvent ratio % using UAE in sunflower oil.

**Fig. 3:**
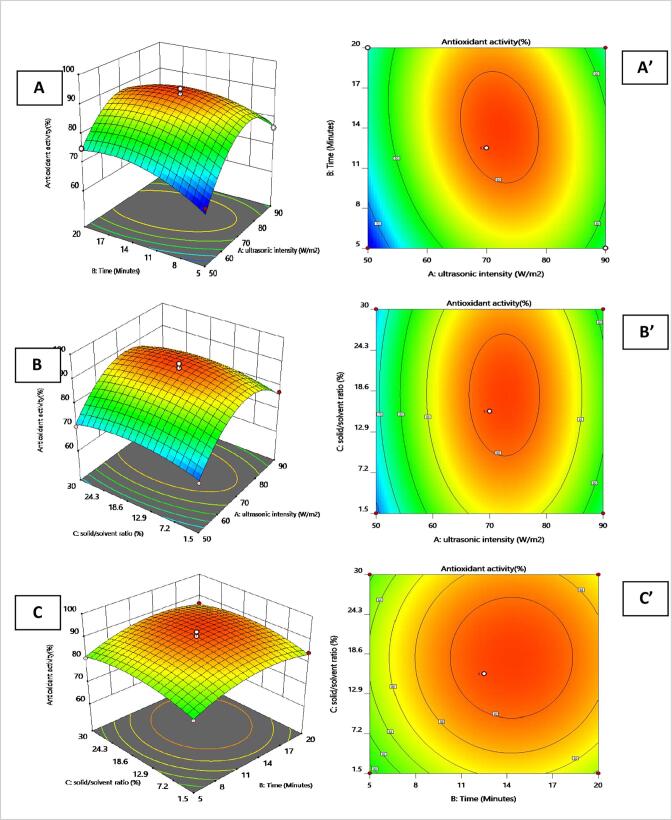
**3D Response plots and 2D contour plots of antioxidant activity of lutein Footnote:**3D response surface plots (A, B, C) and 2D contour plots (A’, B’, C’) of the antioxidant activity of lutein as function of ultrasonic intensity/m^2^, extraction time(minutes) and solid/solvent ratio % using UAE in sunflower oil.

In the current study, the effect of three independent factors (ultrasonic intensity, extraction time, and solid/solvent ratio) on lutein content and antioxidant activity was evaluated using BBD. Response surface plots describe the relationship between each of the two independent variables and the measured responses while keeping the other variable (3rd) constant (Fig. 2, Fig. 3). The ultrasonic intensity was considered a major factor for both responses (lutein content and antioxidant activity) followed by extraction time. Ultrasonic intensity and extraction time were also reported by other studies as the highly influencing parameters affecting the extraction of carotenoids [19; 20]. The amount of lutein extracted got increased with the increase in ultrasonic intensity and time up to a certain level, beyond which a considerable decrease in lutein content (Fig. 2) and antioxidant activity (Fig. 3) can be observed. The highest amount of lutein extracted (21.23 mg/g) from PNG flower petals and its highest antioxidant activity (92.37%) as assumed by BBD came under the following optimum conditions: ultrasonic intensity, 70 W/m^2^; extraction time, 12.5 min; the solid to solvent ratio 15.75 % (Fig. 2). As lutein is the major antioxidant compound present in marigold flower petals, the highest antioxidant activity was observed in the same experimental run, where the maximum output of lutein was extracted. The highest output of lutein came from experiment 1 of Table 1. The experimental and predicted values of lutein extracted and its antioxidant activity is shown in Table 1. The below mentioned equation consists of the fitted prediction model explaining the correlation between the lutein content and the tested independent variables:

**Amount of lutein (mg/g) =** -88.31 + 2.47 × A + 2.45 × B + 0.40 × C −0.003 × A × B + 0.0023 × A × C −0.0012 × B × C + -0.017 × A^2^ −0.078 × B^2^ −0.016 × C^2.^ (7).

Where A represents ultrasonic intensity (W/m^2^), B represents extraction time (minutes) and C represents solid to solvent ratio % (w/v).

#### 3.2.1 Effect of ultrasound intensity on lutein content

In all the tested runs, the amount of lutein was dependent on the intensity and show an increasing trend with extended ultrasonic intensity. The study showed that the highest amount of lutein was extracted at high ultrasonic powers upto 70 W/m^2^. The reason behind this may be enhanced cavitation which increased the surface area of solid and thus allows higher penetration of solvent into the powdered PNG petals[13]. According to Zhang et al. (2008), the higher intensity of ultrasound results in the development of high temperature and pressure inside the bubbles. After a short period, bubbles collapse and result in the generation of intense waves and a highspeed jet, which disrupts the cell walls and enhances the solvent penetration into exposed tissues. This disruption then ensures the enhanced product release into the solvent system[30]. However, increasing the ultrasonic intensity beyond 70 W/m^2^ causes a reduction in lutein content due to the possible degradation of carotenoids at higher power levels. Higher ultrasonic intensity results in the generation of hydroxyl radicals during the process of cavitation, which in turn causes considerable degradation of carotenoids[31]. The results reported from this study were in good agreement to previous studies where increase in ultrasonic intensity upto a particular limit leads to increase in extraction yield of mandarin peel(tian et al 2013) and pomegranate carotenoids[20].

#### 3.2.2 Effect of time on lutein content

Time was another key factor along with ultrasonic intensity which majorly influences the amount of lutein extracted from PNG petals. The efficient extraction time for achieving maximum lutein content was up to 12.5 min. The amount of lutein obtained by UAE as a function of time can be explained by a two-stage mechanism process that is washing stage and slow extraction stage[32]. The first stage of washing, which lasts up to 10 min is characterized by its rapid rate and includes penetration of the solvent into the tissues which subsequently leads to leaching of soluble constituents into the extracting solvent. In this step concentration of lutein in the solvent got increased as this stage accounts for almost 90% of the total carotenoids extracted. The second stage called the slow extraction stage is where the solute got transferred through the porous structure of the solid matrix into the solvent by diffusion[33]. Thus, the ultrasonic waves had a major impact on lutein extraction during the first stage. Moreover, as the cell walls of PNG petals got disintegrated, leaching out of impurities (insoluble materials) begin and remain suspended in the extract, resulting in decreased permeability of solvent into the tissues[34]. Similar findings were also reported by [30], [13] during the UAE of flaxseed oil and carrot carotenoids respectively. It has also been reported that chances of re-adsorption of target components into the ruptured cell materials prevail due to their increased surface areas, lowering the amount of desirable product [35]. Longer extraction time will also result in a reduced amount of lutein because of the possibility of oxidative decomposition. It is reported that ultrasound irradiation for an extended period imposes a negative influence on some active products leading to their chemical decomposition [20].

#### 3.2.3 Effect of solid to solvent ratio on lutein content

According to the mass transfer mechanism, a higher ratio of solid/solvent generally create a greater gradient in concentration throughout the diffusion process from the solid matrix into the solvent, leading to enhanced carotenoid content in the extract. By increasing the solid/ solvent ratio, the chances of carotenoids to come in interaction with extracting fluid increases thus increasing the extraction efficiency[36]. It is also reported that by increasing the solid/solvent ratio, the spontaneity of the extraction is increased as Gibbs free energy (Δ*G*°) becomes more negative with higher content of liquid in the extraction solution [37]. In this study, the greater lutein content was attained at a solid/solvent ratio of 0.15. Further increase in the ratio i.e., above 0.15 shows a lesser increase in the amount of lutein extracted. This may be due to the reason that at this ratio, solution equilibrium has been attained and further addition of solvent renders no extra advantage in the amount of lutein extracted. This was consistent with the findings of [38], [20] who reported that higher extraction yield of phenolics from Mangifera pajang and pomegranate waste was obtained at a particular solid/solvent ratio and any further increase in the ratio does not impart any difference.

The highest free lutein content (21.23 mg/g) reported in this study, is greater than as reported by previous studies for lutein extraction from marigold petals. For instance, the highest lutein content of 10.3 mg/g extracted from dry marigold petals utilising supercritical fluid extraction with soyabean oil as a co-solvent was reported by Ma et al., 2008 [39]. A more recent study reported that the amount of lutein extracted from marigold petals using edible oil was 1.9 times higher than that extracted by using acetone and almost equal to that of extracted by hexane [11]. Higher content of lutein (20.71 mg /g db of marigold flower petals) was reported while extracting and de-esterifying lutein simultaneously using the mixture of liquefied dimethyl ether (DME), KOH, and EtOH [4]. Similarly, the highest amount of lycopene content from tomato waste was reported using a combination of green UAE along with sunflower oil as an extraction solvent [19]. Thus, it can be concluded that ultrasound coupled with edible oil can be thus used as an efficient extraction method both in yield and protective effectiveness of oil against carotenoid degradation. This Green method has been reported to extract carotenoids effectively.

#### 3.2.4 Effect of process parameters on antioxidant activity

The antioxidant activity of the experimental runs was evaluated by DPPH assay. The polynomial equation (eq 8) explains the correlation between the antioxidant activity and the tested independent variables:

**Antioxidant activity (%)** = -125.37 + 5.12 × A + 3.67 × B + 0.67 × C −0.018 × A × B −0.00042 × A × C + 0.00011 × B × C −0.033 × A^2^ −0.082 × B^2^ −0.017 × C^2.^ (8).

Where A represents ultrasonic intensity (W/m^2^), B represents extraction time (minutes) and C represents solid to solvent ratio % (w/v).

According to the optimized results, the highest antioxidant activity of 92.87% was reported at optimum conditions of ultrasonic intensity:70 W/m^2^, extraction time: 12.5 min, and solid to solvent ratio:15.75% pertaining to conditions where the maximum amount of lutein was extracted (experiment 8 of Table 1). Ultrasonic intensity followed by extraction time were the two highly influencing parameters on antioxidant activity of marigold petals similar to that of lutein content extracted. The results were consistent with the study of [40] while extracting polyphenols from olive pomace and [41], while extracting functional compound from makiang seed, where they reported ultrasound power and time were two major factors affecting antioxidant activity. The increased antioxidant activity of UAE lutein may be attributed to the increased extraction of antioxidant rich bioactive constueints from the PNG petal matrix due to cavitation effect of ultrasonic waves[42].

### 3.3 Comparative analysis of UAE and CSE

The optimized results of UAE for lutein employing sunflower oil as an extraction solvent was compared with the CSE. It was observed that the maximum amount of lutein extracted using UAE was about 21.23 mg/g of dry weight in only 12.50 min, significantly higher than that of CSE. However, a lesser concentration of lutein (9.5 mg/g dw) was extracted using the solvent extraction technique in a much-extended time of 24 hr. This trend was consistent with previous studies reporting a 32% (Ordóñez-Santos et al.,2021) and 22%[19] increase in extraction efficiency in samples subjected to ultrasound assisted extraction. Thus, from this study, it can be concluded that ultrasonic-assisted extraction with edible oil can enhance the concentration of lutein while using shorter extraction times and preventing the use of toxic solvent.

The comparative analysis of UAE extract and CSE extract of PNG petal revealed that the former possesses higher TFs than the latter due to the lipophilic nature of oil in which carotenoids are highly soluble. In addition, increased extraction of polyphenols due to cavitation production by ultrasound waves is also the cause of higher flavonoid content in UAE extract. UAE extract shows a TF content of 38.94QE mg/g extract compared to 27.28QE mg/g in CSE. These results were consistent with the study of [43] where they reported a higher content of flavonoid compounds extracted from grapefruit waste by UAE method(24–36 mg/g dw) as compared to CSE (18–28 mg/g dw).

The antioxidant activity of lutein extracted under optimized conditions was further investigated by FRAP and ABTS assay and was compared with the conventionally extracted lutein. UAE optimized lutein show a higher value of FRAP i.e., 0.95 mM TE/g dw of PNG petal as compared to CSE lutein (0.73 mM TE/g). A similar trend was observed for ABTS assay where it was observed that for UAE lutein higher value was displayed (0.74 mM TE/g) as compared to CSE lutein (0.52 mM TE/g) of dry PNG petal. Similarly, (Castaneda et al., 2021)[42] reported that seed extracts of mango showed a higher DPPH, FRAP and ABTS value when extracted by UAE as compared to CSE. The reason was attributed to higher extraction of polyphenolic compounds on application of ultrasound thus increasing its antioxidant activity. In contrast, mango peel extract obtained by UAE showed a lower antioxidant activity as compared to CSE extract, possibly due to degradation of phenolic compounds by the formation of H• and OH• radicals in the medium. Therefore, shorter extraction time and reduced intensity are suggested to minimize the oxidation of bioactive compounds and preserve their antioxidant potential.

### 3.4 Effect of ultrasound on extraction solvent

Ultrasound treatment has been found to cause oxidative deterioration of edible oils, probably due to the cavitation effect which causes structural and functional modifications in the oils up to the point of oxidation. This oxidation got enhanced when ultrasound cavitation is combined with some naturally occurring pro-oxidants like copper in the edible oils [44]. In our study a small difference in untreated oil and ultrasound treated oils at optimum condition was observed depicting that ultrasound does not significantly degrade the quality of oil. Acid value, peroxide value, p-anisidine value and conjugated diene and triene for control and treated oils were 0.35,2.97,5.41,0.92,0.83 and 0.41, 6.12, 3.36, 1.02,0.95 respectively. From these values, it can be concluded that no significant oil deterioration was found in ultrasound-treated oils. The results were in line with the previous studies where no significant difference in oxidative stability was observed between ultrasound treated oil and control oils[20], [37].

### 3.5 FTIR-Assay

**Fig. 4:**
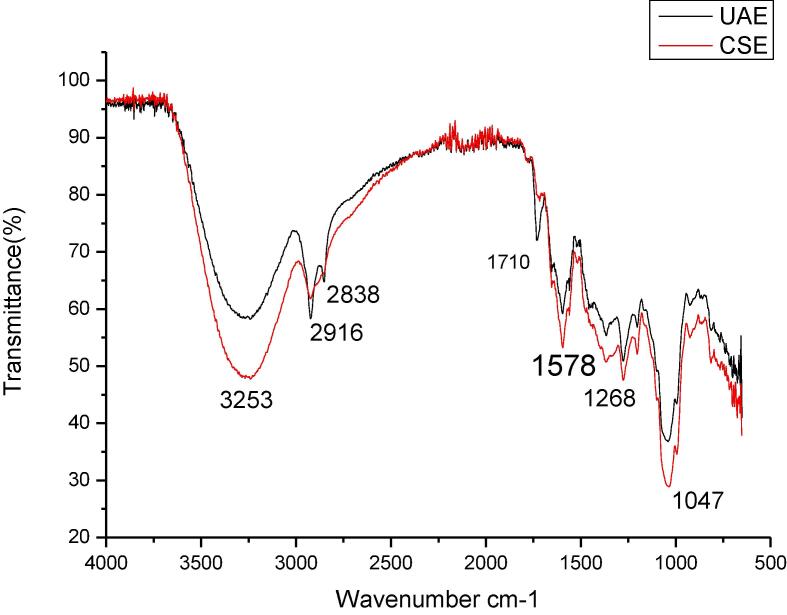
**FTIR spectrum of lutein extracted by UAE and CSE Footnote:** UAE represents lutein extracted by ultrasound and CSE represents lutein extracted by conventional solvent extraction.

The FTIR spectrum of extracted lutein was conducted to identify and assess the impact of ultrasound on functional groups of lutein (Fig. 4). The prominent peaks of lutein were detected at 3253, 2928, 1588, 1269, 1018 cm^−1^. Peaks at 2928 and 3253 represent the -C–H /=C–H stretch vibrations. Bands at 1588 refer to the C = O stretching vibration of ester groups[45]. Similar peaks were identified for both the extracts however ultrasound extracted lutein show an increased percentage transmittance as compared to CSE lutein. This may be because of the possible conversion of functional groups like ester group and carboxylic group on the application of ultrasound rays [46]. In conclusion, exposing raw material to the ultrasonic waves does not affect the position and functionality of the extracted bioactive compound as reported previously [19].

### 3.6 Effect of lutein on oxidative stability of sunflower oil

**Fig. 5:**
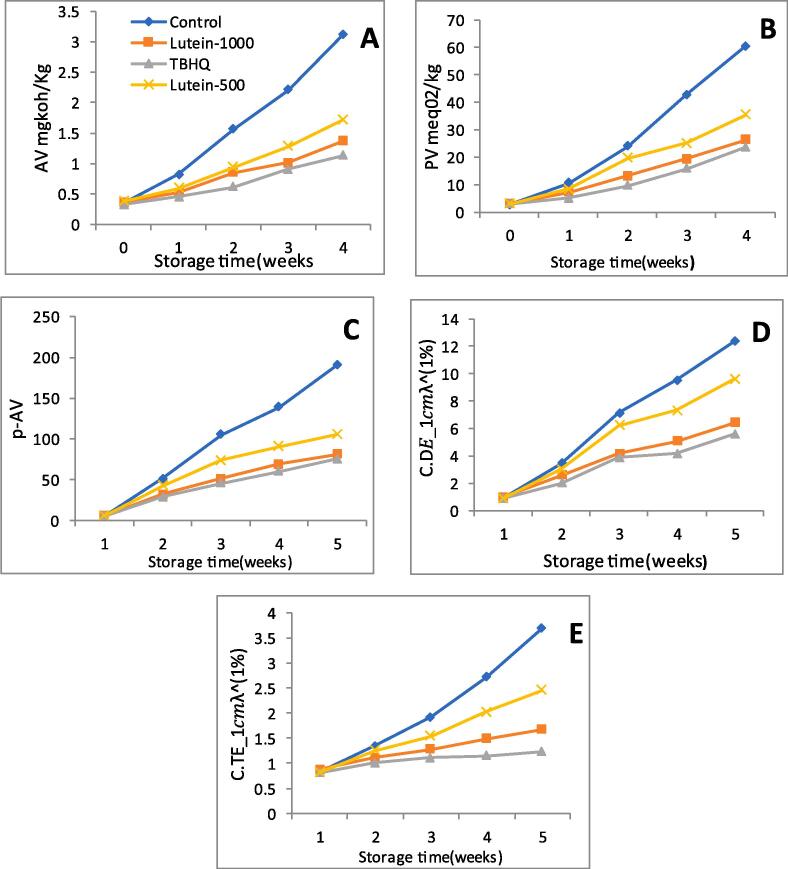
**Oxidative stability of sunflower oil during accelerated storage conditions(A) Acid value(B) peroxide value(C) Para-anisidine value(D) Conjugated Diene(E) conjugated triene Footnote:** Where Lutein-1000 and lutein-500 represents lutein added at a concentration of 1000 ppm and 500 ppm respectively.

The Impact of lutein extract on the oxidative stability of sunflower oils during 4 weeks of accelerated storage conditions is shown in (Fig. 5). From the figure, it can be depicted that lutein at a higher concentration of 1000 ppm presents great oxidative stability to the oil against thermal destruction during accelerated storage. The results of lutein at this concentration were equal or comparable to TBHQ. Acid value measures the extent of deterioration in oils that occurred due to hydrolysis of triglycerides and the decomposition of hydroperoxides by lipase and other actions such as heat and light. It is considered one of the significant parameters to determine deteriorative changes in oil during storage. Sunflower oil containing lutein extract exhibited a lesser increase in acid value in comparison to control oil during 4 weeks of accelerated storage. Peroxide value indicates the formation of primary oxidation products (peroxides and hydroperoxides) due to oxidative degradation [47]. Control sunflower oil shows a peroxide value of 2.97 and 60.6 at zero and 4 weeks of storage. However, antioxidant-rich oils show a lesser amount of PV towards the end of 4 weeks of storage at 50 °C. P-AV determines the degree of formation of products (aldehyde carbonyl compounds) during the secondary lipid oxidation process. The results from our study concluded that secondary oxidation products increase with an extended storage period for both control oils and antioxidant-rich oils, however, in the latter case the increase was considerably lower. Change in absorbance at 232 and 276 nm is related to change in primary and secondary oxidations pertaining to conjugated dienes and conjugated trienes respectively. During the period of storage under accelerated conditions, oxidative degradation of PUFA occurs converting non-conjugated double bonds into conjugated dienes [48]. A drastic increase in conjugated dienes of control oil during the 4-week storage period was detected. However, this change was significantly lower in antioxidant incorporated sunflower oil.

In all the parameters studied, Lutein extract added to sunflower oil at a concentration of 1000 ppm shows antioxidant activity comparable to that of TBHQ (Fig. 5), confirming the great antioxidant potential of lutein. However, at a low concentration of 500 ppm, lutein was not effective against thermal degradation. These findings were similar to previously reported studies, where essential oil of *Coriandrum sativum* L [48] and carnosic acid [47] enhances the oxidative stability of sunflower oil like that of synthetic antioxidant during accelerated storage. Thus, lutein can be used as a potential antioxidant in vegetable oils as it exhibits a protective effect against the thermal oxidation of sunflower oil in accelerated conditions of storage.

## 4 Conclusion

A combination of two green chemistry techniques viz ultrasonication and edible oils were exploited for lutein extraction from PNG petals. The extraction process was optimized using BBD for the effect of 3 independent variables, including ultrasonic intensity, extraction period, and solid/solvent ratio on 2 measured responses i.e., lutein content and antioxidant activity. The highest amount of lutein was extracted under optimized conditions of UAE with an ultrasonic intensity of 70 W/m^2^, extraction time of 12.5 min, and a solid to solvent ratio of 15.75%. When compared with conventional extraction technique (9.18 mg/g), the amount of lutein extracted using UAE (21.23 mg/g) and antioxidant activity (92.7%) was far greater depicting the excellent potential of ultrasound for the extraction of carotenoids. Furthermore, treatment of oils with ultrasound under optimum conditions does not lead to significant oxidative degradation of oils. Sunflower oils incorporated with antioxidants during accelerated storage conditions for 4 weeks shows good oxidative stability as compared to control. Thus, lutein extracted from marigold flower can be used as an effective substituent of toxic synthetic antioxidants in conferring the oxidative stability to edible oils.

## Declaration of Competing Interest

The authors declare that they have no known competing financial interests or personal relationships that could have appeared to influence the work reported in this paper.
