# Supplementary material for: Green extraction of lutein from marigold flower petals, process optimization and its potential to improve the oxidative stability of sunflower oil
Source: Ultrason Sonochem. 2022 Apr 1;85:105994. doi: 10.1016/j.ultsonch.2022.105994 (PMC8980490; doi:10.1016/j.ultsonch.2022.105994)
Supplement: Supplementary data 1 [file mmc1.docx]

Supplementary Table: Experimental domain with coded values of independent variable used in Box-Behnken Design

| **Independent variable** | **Unit** | **Symbol** | **Levels** | | |
| --- | --- | --- | --- | --- | --- |
|  |  |  | -1 | 0 | +1 |
| Ultrasonic power | watt | A | 50 | 70 | 90 |
| Time | minute | B | 5 | 12.5 | 20 |
| Solid to solvent ratio | % (w/v) | C | 1.5 | 15.75 | 30 |
